# Assessment of corticospinal tract damage and cytokines response in early and late stages of acute unilateral brainstem infarction patients

**DOI:** 10.3389/fimmu.2024.1504626

**Published:** 2024-11-29

**Authors:** Mengye Shi, Huiyou Chen, Xiaojiao Ci, Wen Geng, Xinyang Qi, Yuchen Chen, Xindao Yin

**Affiliations:** ^1^ Department of Radiology, Nanjing First Hospital, Nanjing Medical University, Nanjing, China; ^2^ Department of Neurology, Affiliated Nanjing Brain Hospital, Nanjing Medical University, Nanjing, China

**Keywords:** acute brainstem infarction, cytokines, corticospinal tract (CST), probabilistic tractography, fractional anisotropy (FA)

## Abstract

**Background:**

Acute brainstem infarction is associated with high morbidity and mortality, the integrity of corticospinal tract (CST) detected via diffusion tensor imaging (DTI) can assist in predicting the motor recovery of the patients. In addition to the damage caused by ischemia and reperfusion, sterile inflammation also contributes to the brain injury after stroke. However, the changes in CST integrity detected by DTI in acute brainstem infarction have yet to be fully elucidated, and it is still unclear whether sterile inflammation can cause damage to the CST.

**Methods:**

In this study, the acute brainstem infarction patients in the early (EABI patients, n = 19) and late (LABI patients, n = 21) stages and healthy controls (HCs, n = 22) are employed. The probabilistic tractography technique was used and the fractional anisotropy (FA) value was chosen to evaluated the integrity of the CST, the IL-6, IL-10, IL-17, IL-1β, and tumor necrosis factor (TNF)-α levels in the plasma are measured to evaluate the level of inflammation.

**Results:**

Compared to the HCs (F = 13.634, p _ANOVA_ < 0.001), the CST FA values on the infarcted side were abnormally elevated in EABI patients (p _LSD_ = 0.003), and decreased in LABI patients (p _LSD_ = 0.034). The levels of IL-6 (F = 12.311, p _ANOVA_ < 0.001, EABI vs HCs: p _LSD_ < 0.001, LABI vs HCs: p _LSD_ < 0.001), IL-10 (F = 11.329, p _ANOVA_ < 0.001, EABI vs HCs: p _LSD_ < 0.001, LABI vs HCs: p _LSD_ = 0.017) and IL-1β (F = 15.986, p _ANOVA_ < 0.001, EABI vs HCs: p _LSD_ < 0.001, LABI vs HCs: p _LSD_ < 0.001) were increased in both EABI and LABI patients, while the IL-17 levels were elevated only in LABI patients (F = 4.258, p _ANOVA_ = 0.019, LABI vs HCs: p _LSD_ = 0.027). Among these cytokines, the increased IL-6 (r = 0.663, p = 0.002) and IL-1β (r = 0.615, p = 0.005) levels of EABI patients might be related to the elevated CST FA values, while the increased IL-17 (r = -0.599, p = 0.004) levels of LABI patients might contribute to the decrease of the CST FA values.

**Conclusion:**

Our study reveals that the increased CST FA values in EABI patients may include signals generated by the immune cells which move along the CST. The sterile inflammation may contribute to the impairment of CST integrity in LABI patients.

## 1 Introduction

Acute brainstem infarction is associated with high morbidity and mortality, accounting for about 10% of all acute ischemic strokes (1). Understanding the dynamic changes in post-stroke recovery following brainstem infarction is crucial. Previous studies found that the integrity of corticospinal tract (CST), which is fundamental to motor control, is a significant determinant in cerebral infarction (2). In subacute and chronic ischemic stroke patients, CST damage detected via diffusion tensor imaging (DTI) correlates with clinical outcome (2–5). Fractional anisotropy (FA), derived from DTI, serves as an imaging marker that quantifies the organization and integrity of white matter tracts (6). Some studies have reported a significant decrease in the FA value of the CST in acute ischemic stroke patients (6–8), and further investigation suggested that the decrease in FA might assist in predicting the motor recovery of the patients (6, 9, 10). However, there are few studies have focused on the integrity of CST in acute brainstem infarction patients. By using the diffusion kurtosis imaging (DKI) approach, our research group has reported significant differences in the DKI metrics of CST between the infarcted side and the contralateral side in acute brainstem infarction, and there are also significant differences between the infarcted side of patients and the corresponding side of healthy controls (HCs) (11). Nonetheless, the changes in CST integrity detected by DTI in acute brainstem infarction have yet to be fully elucidated.

Besides the direct brain injury resulting from ischemia and reperfusion after stroke, there is increasing evidence that rapidly evolving sterile inflammation plays a significant role in brain impairment (12). Recent studies have revealed that acute cerebral infarction can disrupt the blood-brain barrier, leading to the generation of endogenous signaling and peripheral antigen activation (13, 14). Subsequently, the innate and adaptive immune responses are activated and the peripheral immune cells infiltrate the site of injury, which lead to full-blown neuroinflammation (15). Within the first 24 hours after ischemic stroke, microglia as the first line of immune defense are the first immune cells to be activated (16). Microglia are induced to aggregate within minutes, remove cellular debris and release inflammatory mediators and cytokines, such as interleukin (IL)-10, IL-6, IL-1β (15, 17). Then T cells are recruited by the inflammatory mediators, and differentiate toward two distinct subpopulations: pro-inflammatory and anti-inflammatory cells (15, 18). The anti-inflammatory cells may attenuate inflammatory responses via secretion of cytokines, such as IL-10, while pro-inflammatory cells may product cytokines such as IL-17, tumor necrosis factor (TNF)-α that can damage neural tissue (12, 15). Meanwhile, the peripheral blood immune cells are also recruited by the inflammatory mediators and migrate into the brain within 24 hours (12). The level of the peripheral blood immune cells in the brain peaks at 3 days after stroke, and persist until day 7 after stroke (19). These cells interact with the neural tissue and secrete cytokines such as IL-6, IL-1β, TNF-α, which trigger a secondary neuroimmune response (20, 21). Nevertheless, the dynamic changes in cytokine levels reflecting the level of inflammation in the early and late stages of acute brainstem infarction remain poorly understood, and it is still unclear whether the sterile inflammation can cause damage to the CST.

This study aims to investigate the influence of different cytokines levels on CST integrity in patients with acute brainstem infarction in the early and late stages. First, based on the timing and intensity of the immune response, we divided patients with acute brainstem infarction into two groups: the early acute brainstem infarction patients (EABI patients, within 24 hours after stroke onset) and the late acute brainstem infarction patients (LABI patients, 3 to 7 days after stroke onset). We then chose IL-6, IL-10, IL-17, IL-1β, and TNF-α as indicators to measure the level of inflammation, the probabilistic tractography technique was employed and the FA value was used to evaluated the integrity of the CST in the infarcted side. Finally, we explored the impact of sterile inflammation on the integrity of the CST by comparing the differences in cytokine levels and FA values among the EABI patients, LABI patients and HCs.

## 2 Methods

### 2.1 Participants

A total of 19 left-sided EABI patients and 21 left-sided LABI patients from the Nanjing First Hospital Department of Radiology and 22 HCs were recruited between May 2017 to January 2020 (aged between 40 and 80 years, all right-handed and with at least 6 years of education). Both groups were age, gender, and education well-matched. The inclusion criteria for patients were as follows: (1) between 40 and 80 years of age, (2) right-handedness, (3) first-onset ischemic stroke only in the left brain stem, (4) not received any treatment related to acute cerebral infarction before undergoing MRI scans and blood sampling. Exclusion criteria for all subjects were as follows: (a) a contraindication for MRI (pacemaker, metallic foreign bodies, and severe claustrophobia), (b) severe quadriplegia, (c) a history of neurological and psychiatric disorders, (d) paticipants with any signs of infection.

This study was approved by the Research Ethics Committee of the Affiliated Nanjing Hospital, Nanjing Medical University. Written informed consent was acquired from all subjects. For patients with acute unilateral brainstem stroke, global functional deficits were assessed using National Institutes of Health Stroke Scale (NIHSS).

### 2.2 MRI acquisition

The participants were scanned using a 3.0 T MRI scanner (Ingenia, Philips Medical Systems, Netherlands), with an 8-channel receiver array head coil. Subjects lay supine with their head fixed by foam pads and a belt to minimize head motion. And earplugs were used to reduce scanner noise by approximately 32 dB according to the manufacture’s data sheet. The subjects were instructed to lie quietly and keep their eyes closed but not to fall asleep, not to think of anything special, and to avoid head motion during the functional MRI. Structural images were obtained using a three-dimensional turbo fast echo T1WI sequence using the following parameters: repetition time (TR)/echo time (TE) =8.1/3.7 ms; slices =170; thickness =1 mm; gap =0 mm; flip angle =8°; acquisition matrix =256×256; field of view (FOV) =256×256 mm2. DTI employed the following parameters: TR =3,000 ms; TE =1,000 ms; slices =55; thickness =2.5 mm; gap =0; flip angle =90°; acquisition matrix =120×177; and FOV =240 mm × 240 mm; 32 volumes with diffusion gradients along 32 nonlinear directions [b=1,000 s/mm2] and 1 volume without diffusion weighting [b=0 s/mm2]. The DTI sequence took 11 min and 9 s. The SENSE is used for parallel imaging.

### 2.3 Data preprocessing

DTI data were pre-processed using the FSL Brain Extraction Tool for skull stripping and the FSL Diffusion Toolkit to minimize eddy current distortion effects and for registration of the diffusion volumes (22).

### 2.4 Probabilistic tractography

**Figure 1:**
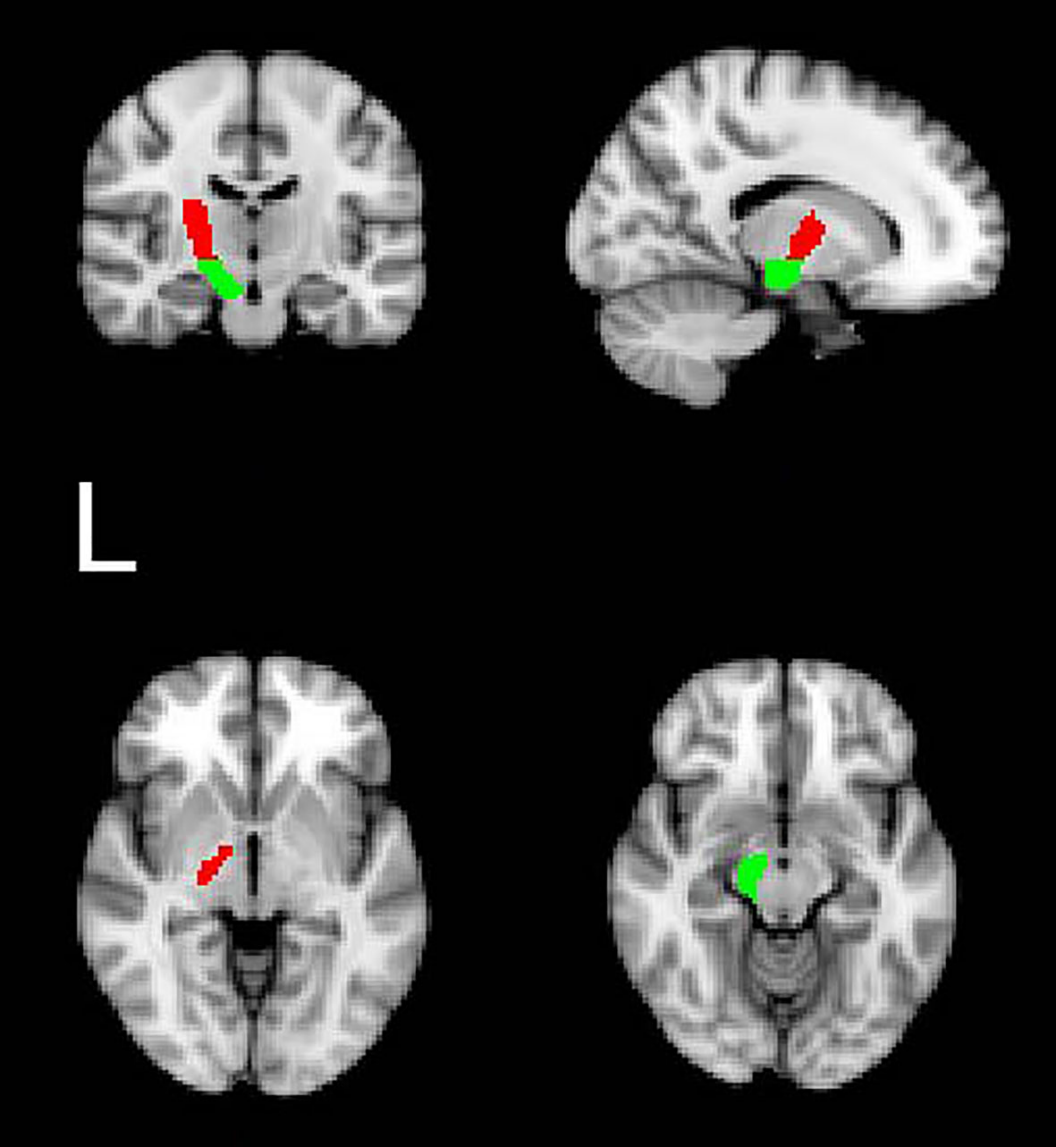
The probabilistic tractography seed regions: examples of regions of interest in the standard space (left PLIC: red; left CP: green).

**Figure 2:**
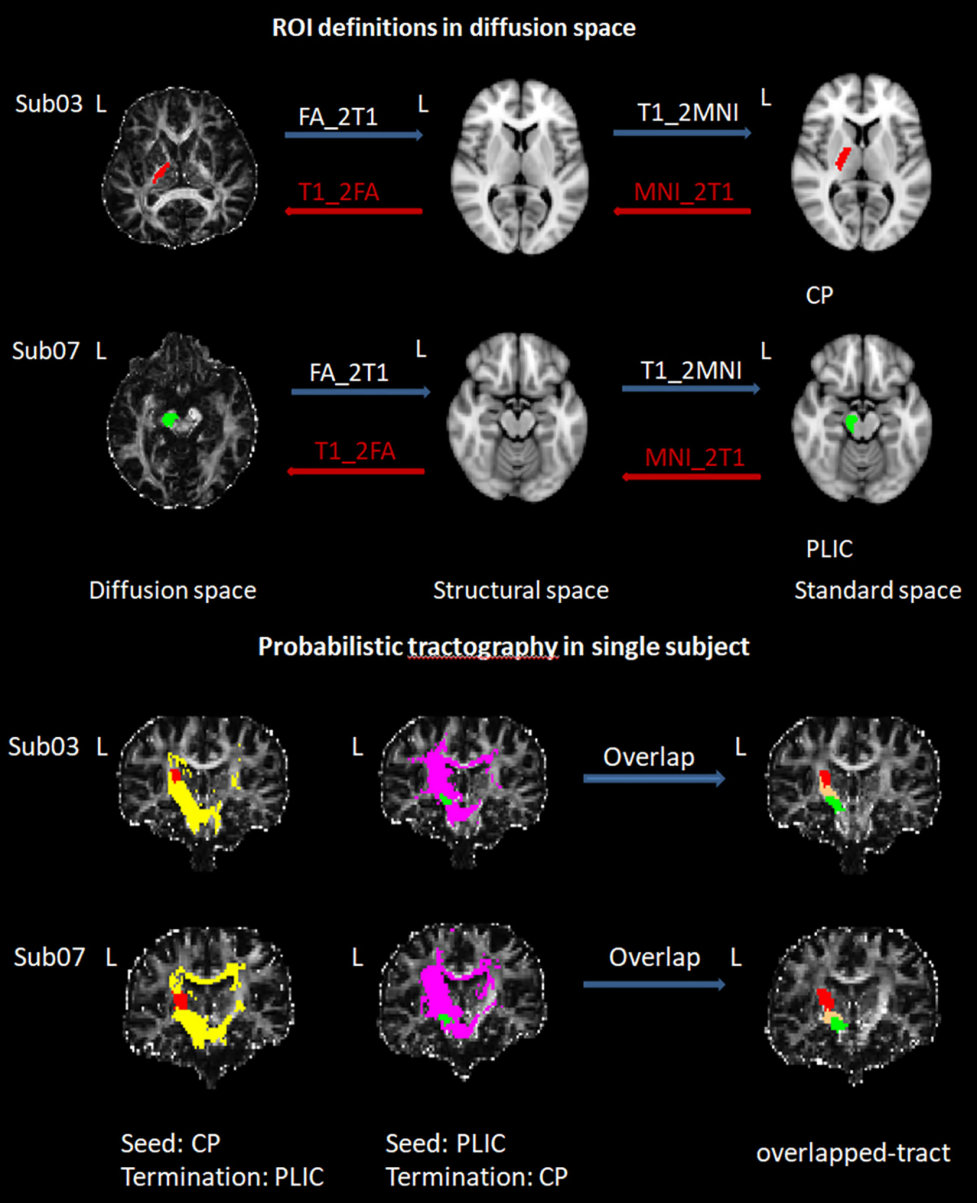
Regions of interest definition in diffusion space and DTI Probabilistic tractography of left CST fiber. The ROIs from MNI space were transformed into native diffusion space. Initially, the DTI images were rigidly registered to the individual T1-weighted image using FSL’s Linear Image Registration Tool (FLIRT), employing mutual information as the cost function to generate the FA_2T1 matrix. The individual T1 image was then normalized to MNI space through both linear (FLIRT) and nonlinear registration (FNIRT, FSL’s Nonlinear Image Registration Tool). Subsequently, the co-registered DTI image in structural space was warped using the transformation field obtained from the T1 to MNI normalization. The transformation matrix (FA_2T1) and warp-fields (T1_2MNI warp) were inverted using *inverse* and *invwarp* command, respectively. These inverse transformations were applied to the ROIs in MNI space to obtain the corresponding ROIs in native diffusion space. We used the BEDPOSTX (Bayesian Estimation of Diffusion Parameters Obtained using Sampling Techniques) to calculate the distribution of fiber orientations at each brain voxel. To ensure that only the white matter fibers were tracked, tractography for each hemisphere was conducted twice, once with the left CP as the seed mask and the left PLIC as the termination mask, and vice versa (i.e., the left PLIC = seed mask; the left CP = termination mask, the left CST tract). For each hemisphere and species, we applied a threshold to include only those voxels that received at least 3.08 × 10^-5 percent of the total streamlines emitted from the ROI masks used to trace each tract (a total of 25,000 times the number of voxels in the mask). Furthermore, tracts in diffusion space were binarized and combined by overlapping operation (overlapped-tract) for each subject. Finally, we masked diffusion maps with the binarized overlapped-tracts to compute the mean value of FA within the final-tract mask in diffusion space.

According to a previous study, we utilized the Bayesian Estimation of Diffusion Parameters Obtained using Sampling Techniques (BEDPOSTX) command to estimate the distribution of fiber orientations in each brain voxel (23). The seed regions, defined according to the origin of the CST, included the cerebral peduncle (CP) and the posterior limb of the internal capsule (PLIC, **Figure 1**) (24, 25). These regions were extracted from the JHU ICBM-DTI-81 White-Matter Labels. To ensure that only the white matter fibers were tracked, tractography for each hemisphere was conducted twice, once with the left CP as the seed mask and the left PLIC as the termination mask, and vice versa (i.e., the left PLIC = seed mask; the left CP = termination mask, the left CST tract). For each hemisphere and species, we applied a threshold to include only those voxels that received at least 3.08 × 10^-5 percent of the total streamlines emitted from the ROI masks used to trace each tract (a total of 25,000 times the number of voxels in the mask) (26). Furthermore, tracts in diffusion space were binarized and combined by overlapping operation (overlapped-tract) for each subject. Finally, we masked diffusion maps with the binarized overlapped-tracts to compute the mean value of FA within the final-tract mask in diffusion space (27). To mitigate contamination from cerebrospinal fluid (CSF) and partial volume effects, we applied a threshold of 0 to 1 to the FA maps (7). Detailed processing steps are illustrated in **Figure 2**.

### 2.5 Biomakers of cytokines

Whole blood samples were collected upon admission. Plasma was then extracted and stored at -80°C. The concentrations of cytokines (IL-6, IL-10, IL-17, TNF-α, and IL-1β) were systematically measured using the 12-Cytokine Detection Kit (Raisecare, Qingdao, China) and analyzed with a Navios flow cytometer (Beckman Coulter, California, USA), in accordance with the manufacturer’s protocol.

### 2.6 Statistical analysis

Data were expressed as mean ± s.e.m. and analyzed using SPSS 20.0 software (SPSS, Inc., Chicago, IL, USA). T-tests, χ2-tests and one-way ANOVA followed by LSD *post hoc* tests were used for data analyses. Then we performed Pearson’s correlation analyses between the FA values and each cytokines levels. P<0.05 was considered to indicate a statistically significant difference.

## 3 Results

### 3.1 Demographic and clinical characteristics

The demographic and clinical characteristics of patients and HCs are summarized in **Table 1**. There were no significant differences in age (F = 1.550, p _ANOVA_ = 0.221), sex (χ2 = 1.358, p = 0.507), or education level (F = 1.351, p _ANOVA_ = 0.267) among the EABI patients, LABI patients and HCs. Additionally, no significant difference was found in the NIHSS scores (t = -0.670, p = 0.507) between the EABI and LABI patients.

**Table 1:** Demographic and clinical characteristics of all subjects.

	EABI (n=19)	LABI (n=21)	HCs (n=22)	*P*
Age (yr)	60.263 ± 1.657	60.333 ± 0.944	57.455 ± 1.374	0.221
Gender (male/female)	12/7	12/9	10/12	0.507
Education (yr)	11.895 ± 0.630	10.905 ± 0.609	10.636 ± 0.444	0.267
NHISS	3.158 ± 0.368	3.619 ± 0.563		0.507
Hypertension (%)	78.9%	85.7%		0.574
Systolic pressure	155.737 ± 5.526	147.286 ± 2.995		0.176
Diastolic pressure	86.158 ± 2.510	86.095 ± 1.953		0.984
Diabetes (%)	57.9%	66.7%		0.567
Smoke (%)	26.3%	28.6%		0.873
Alcohol (%)	21.1%	19.0%		0.120

EABI, early acute brainstem infarction; LABI, late acute brainstem infarction; HCs, healthy controls; NHISS, National Institutes of Health Stroke Scale. Data are expressed as mean ± s.e.m.

### 3.2 Comparison of FA values in CST between patients and HCs

**Figure 3:**
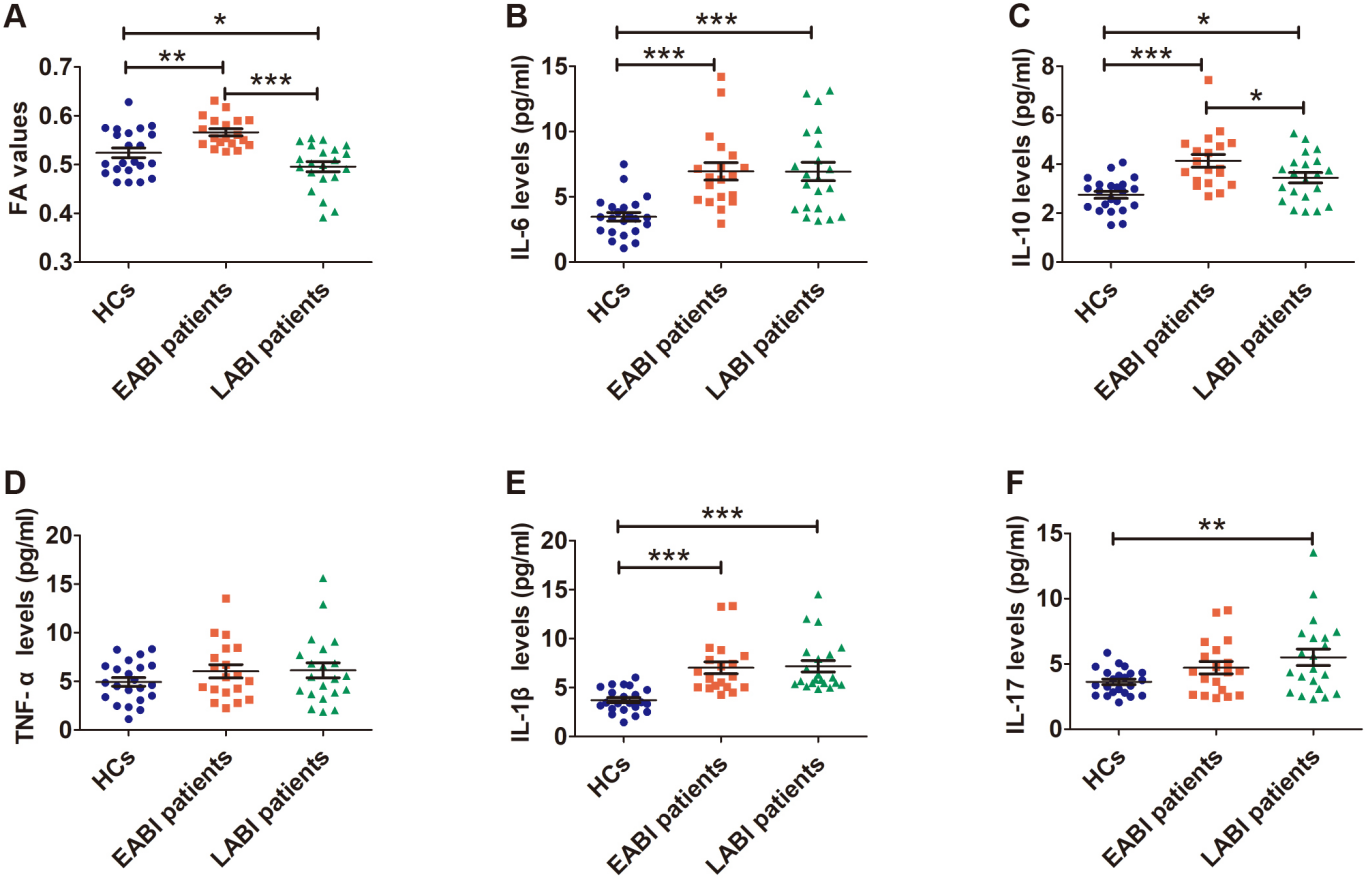
FA values **(A)** and levels of cytokines **(B–F)** in EABI (n=19), LABI (n=21) patients and HCs (n=22). *p<0.05, **p<0.01, ***p<0.001.

The FA values in the CST obtained by the tractography approach on the infarcted side of EABI patients were significantly higher (**Figure 3A**, F = 13.634, p _ANOVA_ < 0.001) than those of LABI patients (p _LSD_ < 0.001) and HCs (p _LSD_ = 0.003), while the FA values of LABI patients were significantly lower than those of EABI patients (p _LSD_ < 0.001) and HCs (p _LSD_ = 0.034).

### 3.3 Comparison of cytokines levels between patients and HCs

The levels of cytokines were measured in plasma from both patients and HCs, the levels of IL-6 (**Figure 3B**, F = 12.311, p _ANOVA_ < 0.001) and IL-1β (**Figure 3E**, F = 15.986, p _ANOVA_ < 0.001) were significantly elevated in EABI (IL-6: p _LSD_ < 0.001, IL-1β: p _LSD_ < 0.001) and LABI (IL-6: p _LSD_ < 0.001, IL-1β: p _LSD_ < 0.001) patients compared to HCs, and there were no differences between EABI and LABI patients (IL-6: p _LSD_ = 0.984, IL-1β: p _LSD_ = 0.8825). Compared to HCs (**Figure 3C**, F = 11.329, p _ANOVA_ < 0.001), the EABI (p _LSD_ < 0.001) and LABI (p _LSD_ = 0.017) patients had higher levels of IL-10, moreover, the EABI patients had higher IL-10 levels than the LABI patients (p _LSD_ = 0.023). The IL-17 levels (**Figure 3F**, F = 4.258, p _ANOVA_ = 0.019) of the LABI patients were significantly higher than that of HCs (p _LSD_ = 0.027), while the IL-17 levels of the EABI patients showed no significant difference compared to the LABI patients (p _LSD_ = 0.683) and HCs (p _LSD_ = 0.131). The results showed no significant differences in the TNF-α levels among the three groups (**Figure 3D**, F = 1.126, p _ANOVA_ = 0.331).

### 3.4 Correlation between cytokines levels and FA values

**Figure 4:**
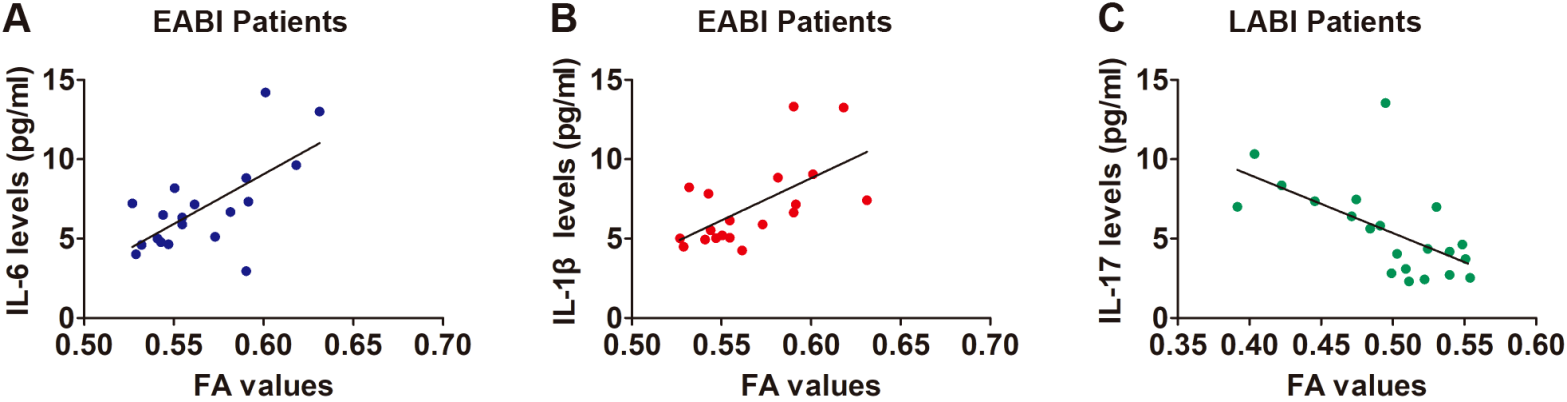
Correlation between the FA values and levels of cytokines. **(A)** A positive correlation between the FA values and IL-6 levels in EABI patients (n=19, r = 0.663, p = 0.002). **(B)** A positive correlation between the FA values and IL-1β levels in EABI patients (n=19, r = 0.615, p = 0.005). **(C)** A negative correlation between the FA values and IL-17 levels in LABI patients (n=22, r = -0.599, p = 0.004).

In EABI patients, the CST FA values on the infarcted side were found to be positively correlated with the levels of IL-6 (**Figure 4A**, r = 0.663, p = 0.002) and IL-1β (**Figure 4B**, r = 0.615, p = 0.005). There was a negative correlation between the FA values and the levels of IL-17 in LABI patients (**Figure 4C**, r = -0.599, p = 0.004).

## 4 Discussion

In the present study, we found that compared to the HCs, the CST FA values on the infarcted side were abnormally elevated in EABI patients, and decreased in LABI patients. The levels of IL-6, IL-10 and IL-1β were increased in both EABI and LABI patients, while the IL-17 levels were elevated only in LABI patients. Among these cytokines, the increased IL-6 and IL-1β levels of EABI patients might be related to the elevated CST FA values, while the increased IL-17 levels of LABI patients might contribute to the decrease of the CST FA values. To the best of our knowledge, this is the first study to investigate the impact of cytokines on the CST FA values in the early and late stages of acute brainstem infarction.

Sterile inflammation is crucially involved in the pathophysiology of the acute damage cascades after ischemic stroke (16, 28). Once ischemic stroke has developed, focal brain ischemia triggers cell death, resulting in the uncontrolled release of intracellular molecules (29). These molecules act as damage-associated molecular patterns (DAMPs) and stimulate sterile inflammation (12, 30). The DAMPs can activate both brain resident cells, adaptive cells and peripheral immune cells (17). As the important resident immune cells in the brain, microglia are activated in hours, and differentiate into M1-like or M2-like subsets (15). The M1-like pro-inflammatory phenotype can secrete cytokines such as IL-6, IL-1β, which may exacerbate the pathology (31). The M2-like phenotype can secrete anti-inflammatory cytokines such as IL-10, which protect the brain tissues (32). Meanwhile, the activated microglia also act as the antigen-presenting cells, which activate the adaptive immunity system (15). T cells are the adaptive cells involved in the sterile inflammation during the acute phase of cerebral ischemia, different types of T cells infiltrate the brain at different times (15). In the first 24 hours after stroke, CD4+ Th1 and Th17 cells, as well as γδT and CD8+ cells are the predominant pro-inflammatory T cells, which secrete cytokines such as IL-17, TNF-α, infiltrate the infarcted area (33). While Treg cells secrete IL-10, which exert anti-inflammatory effects (34). Among these T cells, the levels of Th1 and Th17 cells peak on the 3rd day after stroke and start to decline from the 7th day (35), the number of CD8+ cells also peaks on the 3rd day (36). Moreover, the IL-17 secreted by γδT cells peaks on the 3rd day after stroke (37, 38), while the level of Treg cells do not show significant increasing until the 7th day (15). Neutrophils and inflammatory monocytes are the peripheral immune cells recruited to the injury site (12). These cells migrate into the brain within 1 day after stroke, peaking at the 3rd day (19). They can secrete both anti-inflammatory cytokines such as IL-10, and pro-inflammatory cytokines such as IL-6, IL-1β, TNF-α (12, 21, 39). In summary, the resident cells, adaptive cells and peripheral immune cells are activated within 24 hours after stroke, most of the immune response levels peak on the 3rd day and persist until the 7th day. By detecting the levels of cytokines secreted by the immune cells, we found that both anti-inflammatory (IL-10) and pro-inflammatory (IL-6, IL-1β) cytokines levels are significantly evaluated in the 24 hours after acute brainstem infarction. The increased cytokines levels persist in the late stage of acute brainstem infarction, while the IL-17 level only increased in the late stage. These findings consist with the previous studies and further reveal the dynamic changes of cytokines levels in the acute phase of brainstem infarction.

CST is regarded as the most critical motor pathway in human brain (2, 40). Previous studies have demonstrated that assessment of CST integrity in the chronic phase of stroke is closely associated with motor outcome (4, 5), suggesting that the integrity of CST may be an important indicator for assessing the prognosis of stroke. In the acute stroke, some studies have found that the FA values of the ipsilesional CST are decreased compared to the HCs (6–8). By using the DKI approach, our research group has reported that the FA values of CST on the affected side of the acute brainstem infarction patients are significantly reduced compared to the contralateral side of these patients and the corresponding side of HCs (11). In this study, we further investigated the CST integrity by the probabilistic tractography technique in the EABI and LABI patients. Interestingly, we found that the FA values of the EABI patients are abnormally elevated, and the increased FA values may be related to the increased IL-6 and IL-1β levels. Based on these findings, we proposed a hypothesis that the increased FA values may be caused by the migration of the immune cells. The FA value is the ratio of the anisotropic part of the diffusion to the total value of the diffusion tensor. It reflects the proportion of the anisotropic component within the entire diffusion tensor, with values ranging from 0 to 1. A value of 0 represents maximum isotropic diffusion, such as the diffusion of water molecules in a completely homogeneous medium, while a value of 1 represents maximum anisotropic diffusion under hypothetical conditions. In white matter, the FA value is positively correlated with the integrity of myelin, the density of fibers, and their parallelism. In the first 24 hours after acute brainstem infarction, a lot of adaptive cells and peripheral immune cells are recruited and migrate into the injury site. The movement direction of these cells may be aligned with the orientation of the white matter fibers. Therefore, the measured CST FA values in EABI patients may include signals generated by these immune cells which move along the CST. This may explain why the CST FA values of these patients are higher than those of HCs. In addition, we found that compared to HCs, the CST FA values of the LABI patients are reduced. The decreased FA values are related to the increased IL-17 levels, suggesting the sterile inflammation may contribute to the impairment of CST integrity.

The present study has some limitations. First, the sample size of this study is relatively small, our findings need to be validated in a larger population. Second, this study investigated the differences in cytokines and CST FA values respectively in the EABI and LABI patients. Future research should exam the same acute brainstem infarction patient in early and late stage to confirm these findings.

## 5 Conclusion

In summary, this study observed the changes of cytokines levels and CST FA values in early and late stages of acute brainstem infarction patients, clarified the impact of cytokines levels on the CST FA values in different stages. This provides new insights for future research to investigate the effects of sterile inflammation on CST integrity in acute brainstem infarction, which may help in providing an improved clinical treatment.

## Data Availability

The original contributions presented in the study are included in the article/**Supplementary Material**. Further inquiries can be directed to the corresponding authors.
